# Ontologies for increasing the FAIRness of plant research data

**DOI:** 10.3389/fpls.2023.1279694

**Published:** 2023-11-30

**Authors:** Kathryn Dumschott, Hannah Dörpholz, Marie-Angélique Laporte, Dominik Brilhaus, Andrea Schrader, Björn Usadel, Steffen Neumann, Elizabeth Arnaud, Angela Kranz

**Affiliations:** ^1^ Institute of Bio- and Geosciences (IBG-4: Bioinformatics) & Bioeconomy Science Center (BioSC), CEPLAS, Forschungszentrum Jülich, Jülich, Germany; ^2^ Digital Solutions Team, Digital Inclusion Lever, Bioversity International, Montpellier Office, Montpellier, France; ^3^ Data Science and Management & Cluster of Excellence on Plant Sciences (CEPLAS), Heinrich Heine University Düsseldorf, Düsseldorf, Germany; ^4^ Data Science and Management & Cluster of Excellence on Plant Sciences (CEPLAS), University of Cologne, Cologne, Germany; ^5^ Institute for Biological Data Science & Cluster of Excellence on Plant Sciences (CEPLAS), Faculty of Mathematics and Life Sciences, Heinrich Heine University Düsseldorf, Düsseldorf, Germany; ^6^ Program Center MetaCom, Leibniz Institute of Plant Biochemistry, Halle, Germany; ^7^ German Centre for Integrative Biodiversity Research (iDiv), Halle-Jena-Leipzig, Germany

**Keywords:** data management, metadata, FAIR, ontologies, OBO foundry, DataPLANT, ISA

## Abstract

The importance of improving the FAIRness (findability, accessibility, interoperability, reusability) of research data is undeniable, especially in the face of large, complex datasets currently being produced by omics technologies. Facilitating the integration of a dataset with other types of data increases the likelihood of reuse, and the potential of answering novel research questions. Ontologies are a useful tool for semantically tagging datasets as adding relevant metadata increases the understanding of how data was produced and increases its interoperability. Ontologies provide concepts for a particular domain as well as the relationships between concepts. By tagging data with ontology terms, data becomes both human- and machine- interpretable, allowing for increased reuse and interoperability. However, the task of identifying ontologies relevant to a particular research domain or technology is challenging, especially within the diverse realm of fundamental plant research. In this review, we outline the ontologies most relevant to the fundamental plant sciences and how they can be used to annotate data related to plant-specific experiments within metadata frameworks, such as Investigation-Study-Assay (ISA). We also outline repositories and platforms most useful for identifying applicable ontologies or finding ontology terms.

## 1 Introduction

The field of plant research encompasses a huge range of experimental designs and analytical techniques in order to elucidate the complex, interconnected mechanisms involved in plant systems in a controlled manner, providing insights into the respective mechanisms and facilitating the development of new technologies and strategies for improving crop productivity, disease resistance, and environmental sustainability (Shah and Wu, 2019; Senger et al., 2022; Baekelandt et al., 2023). Documenting the experimental designs and resulting research data ranges from describing the experimental set up to its implementation, from sample characteristics to experimental or environmental factors, and from capturing phenotyping and imaging data to molecular analyses such as genomics, transcriptomics, proteomics and metabolomics data. The size and complexity of such experimental designs and the resulting data challenge good data management practices and the preservation of data. Conversely, some investigations result in scarce amounts of data that may be significant if combined with additional datasets, as long as they are properly preserved. For this reason, the FAIR (findable, accessible, interoperable and reusable) principles were designed to guide data producers to maximize good data management practices (Wilkinson et al., 2016). FAIR data ensures transparency, reproducibility, and interoperability of plant science research, facilitating collaboration among scientists and enhancing the overall quality and impact of research outcomes. This in turn allows scientists to more easily contribute to, and more rapidly adapt to, the development of sustainable solutions for addressing global challenges such as food security and climate change (Mayer et al., 2021; Arend et al., 2022).

One key component of research data management (RDM) is the comprehensive and accurate description of metadata, or data about data. Metadata provides essential information about the context, content, and characteristics of the data, helping researchers to organize, describe, and understand datasets and their production, enabling effective data discovery, sharing, and reuse (Wilkinson et al., 2016). The correct and complete recording of metadata relating to an investigation is especially important for plant research data as environmental conditions can have such a profound influence over the resulting data of sessile organisms (Ćwiek-Kupczyńska et al., 2016).

With the large amounts of data being generated for a single research project, the potential and benefit of reusing and combining datasets to facilitate novel scientific discoveries is becoming ever greater. The challenge lies in the ability to find and integrate relevant datasets from different sources. Metadata is crucial for the correct interpretation of experimental data. Consistency in metadata annotation is important to ensure data is interoperable. It is crucial that datasets are both standardized as well as not only human- but also machine-readable, especially in the plant sciences, where diverse types of data are collected, analyzed and integrated (Shaw et al., 2020; Pommier et al., 2023).

In recent years, ontologies have emerged as a powerful building block, supporting the standardization and harmonization of data annotation in plant sciences and increasing their FAIRness. Ontologies are systematic descriptions of knowledge used to describe a specific domain (Jensen and Bork, 2010). They are composed of a collection of terms as well as the relationships between them, which adds context and structure. Ontologies provide unique identifiers for concepts, making them machine-readable and retrievable. The standardized definitions for terms ensure that metadata tagged with ontology terms is interoperable between researchers.

In addition to ontologies, metadata frameworks are important and widely-used data models for increasing the interoperability and shareability of data (Sansone et al., 2012). Metadata frameworks promote the structuring of data, ensuring it is in a consistent format which allows both data producers and consumers to effectively work with a diverse dataset. Well known examples of metadata frameworks include lightweight Bioschemas (Michel and The Bioschemas Community, 2018) and the ISA metadata framework (Investigation-Study-Assay) (Sansone et al., 2012; González-Beltrán et al., 2014). Implementing metadata frameworks in conjunction with ontologies further facilitates the FAIRness of data.

In recent years, several large-scale efforts aiming to provide services and tools that contribute to increasing the FAIRness of research data have been organized. Examples of such efforts are the German National Research Data Infrastructure (NFDI) (https://www.nfdi.de/; Hartl et al., 2021) and Elixir (https://elixir-europe.org/; Crosswell and Thornton, 2012). The NFDI comprises 26 consortia from different scientific disciplines, many of which offer software that facilitate standardized and comprehensive metadata annotation (Sasse et al., 2022). For example, the plant-focused NFDI DataPLANT consortium offers a metadata annotation tool, Swate, that incorporates ontologies necessary for the annotation of plant-specific experiments (Mühlhaus et al., 2021).

While there is no ‘one size fits all’ approach to correctly annotating data with ontology terms, the task of selecting a specific term or ontology is often daunting and confusing to newcomers due to diverse and scattered resources. To aid researchers in this task, this review will provide an overview of ontologies most relevant to the fundamental plant sciences as well as their role and application in the annotation and integration of plant-specific experiments and how they relate to metadata frameworks, such as ISA. We will outline repositories and platforms most helpful for finding ontologies or ontology terms applicable to the annotation of metadata in the fundamental plant sciences. Finally, we will discuss the importance of community engagement for the interoperability of ontologies and ensuring that ontologies reflect the most recent scientific advancements.

## 2 Ontologies for increased interoperability of research data

**Figure 1:**
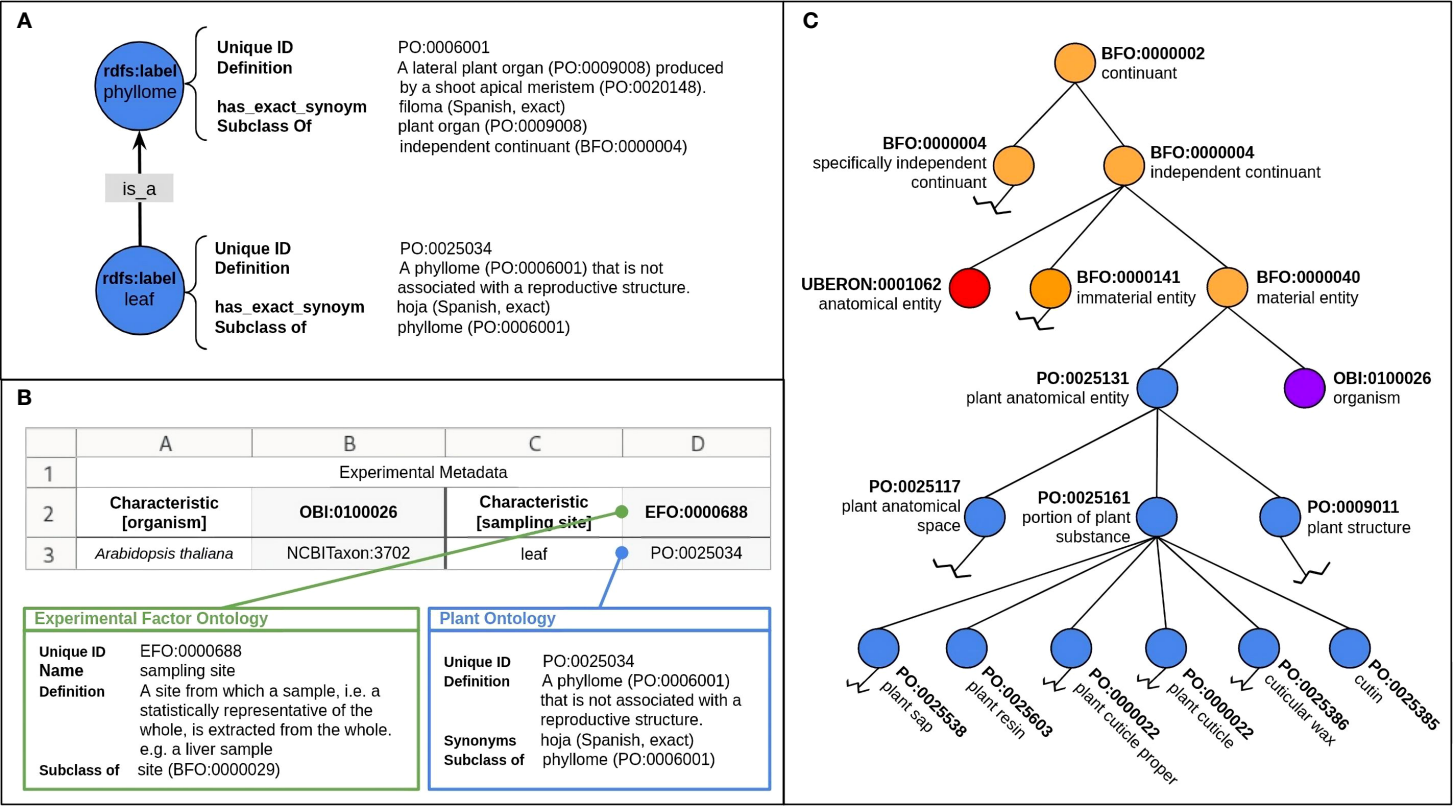
Ontology structure and functions. **(A)** Ontology classes (blue circles) contain information such as a unique identifier, name, definition and synonyms for describing the class. Relations (arrow) connect classes, giving ontologies structure and context. **(B)** Ontologies can import and share terms from other ontologies to enable consistent representation of a term (concept) or domain, increasing the interoperability and standardization of ontologies and the terms they contain. Pictured here is an excerpt of the Plant Ontology (PO, blue nodes) (Walls et al., 2012; Cooper et al., 2013), containing terms from the Basic Formal Ontology (BFO, orange nodes) (Arp et al., 2015), the Uberon multi-species anatomy ontology (UBERON, red nodes) (Mungall et al., 2012) and the Ontology for Biomedical Investigations (OBI, purple nodes) (Brinkman et al., 2010). **(C)** An example of how ontology terms can be incorporated into metadata sheets to ‘tag’ information, facilitating machine-readability and increasing the FAIRness of the data. Columns A and C depict terms and columns B and D depict the corresponding ontology ID, respectively. Row 2 depicts the experiment characteristic and row 3 is the corresponding value.

Ontologies are formal descriptions of knowledge that define concepts or terms and categories within a specific domain, as well as the relationships between them (**Figure 1**) (Gruber, 1993). While structure and semantic language of an ontology facilitate automatic reasoning, ontologies created within the biological sciences often focus on hierarchically describing their concepts and terms, meaning that they can be used as a well-organized controlled vocabulary for a specific domain. In the context of RDM, they are important for the structuring and standardization of data, improving interoperability and facilitating data integration and reuse.

Ontologies need to fulfill certain technical requirements and structures to contribute to standardized metadata annotation. They are made up of two primary components: classes and the relations between them (**Figure 1A**). Classes define general concepts, terms or types of objects within a particular scientific domain. For example, terms such as ‘plant structure’ and ‘abiotic plant exposure’ are concepts that could be necessary to annotate a plant-related experiment. Relations describe how classes are connected and create semantic context within ontologies, giving them (often hierarchical) structure (**Figure 1B**). The most commonly found relation in an ontology is SubClassOf (is_a).

Classes contain information, or annotations, about the particular concept or term, including a human-readable name or label, definition, equivalent terms, and synonyms. Most importantly, ontology classes contain a unique identifier, such as a persistent identifier (PID) or a Uniform Resource Identifier (URI). These are permanent and unique, which allows the term to be permanently identified (Osumi-Sutherland, 2020). An ontology term’s PID can be used to ‘tag’ metadata within a document, thereby making it machine-readable (**Figure 1C**).

Ontologies can be expressed in a number of ways which vary in their human-readability and usage. The most commonly expressed forms within the biological and plant sciences are the Open Biological and Biomedical Ontology (OBO) file format and the Web Ontology Language (OWL) (Antoniou and van Harmelen, 2004; Golbreich et al., 2007). While ontologies vary in terms of content, one nearly ubiquitous characteristic of ontologies, especially within the biomedical and plant sciences, is the open and collaborative mindset with which its developers work to increase interoperability. One way of accomplishing this is to reuse, or import, terms from an existing ontology if the term adequately describes what is needed (Xiang et al., 2010), or else to use import from an upper-level ontology to organize terms at the most general level (**Figure 1C**). Other reasons for importing terms are, for example, commonly used measurement units, or the creation of a very specific application ontology, which may require terms from a related domain ontology to give proper context or structure.

An additional benefit of term reuse and importing is that even if an ontology project runs out of support or funding, proper integration and cross-referencing of terms in other ontologies ensures that their knowledge is not lost. Interoperability can also be increased via community engagement. Contributions from domain experts (researchers) help grow and improve ontologies, keeping them up to date as the science itself develops and scientific discoveries are made. Git technology (Chacon, 2014) and GitHub (https://github.com/) have been transformative for the ontology community for facilitating open source work and version control. Researchers are able to interact with ontology providers to suggest terms and term improvements via the issues tab, and files are transparent and traceable, with changes and updates clearly marked and easily findable.

## 3 Ontology resources

While the benefit of tagging metadata with ontology terms is clear, determining how to select an appropriate ontology or term is often less clear. Over recent years, a number of resources involved with the collection, curation and development of ontologies for particular scientific domains have been developed. These are often good starting points when deciding what term is best for metadata annotation. As they are easily searchable and often give the current developmental status of the ontologies they include, researchers can find the information they need to select a suitable ontology, or ontology term, for their particular data.

Among these is the Open Biological and Biomedical Ontology (OBO) Foundry (https://obofoundry.org/; Smith et al., 2007), a collaborative initiative with the aim of developing a set of interoperable ontologies intended for the biological sciences. The OBO Foundry is a development community with a set of guiding principles that are seen as good practice, working to increase interoperability of ontologies. The principles are improved and refined at regular intervals and many are operational, allowing for easier interpretation and adherence (Jackson et al., 2021). The principles cover all aspects of ontology development, from licensing and formatting to documentation and commitment to collaboration. Ontologies wishing to be accepted into the OBO community are checked against these principles before being accepted. While not a classic ontology repository, the ontologies included in the OBO registry are considered to be adhering to best practices and that terms and relations found within are actively maintained. The registry covers a wide range of ontologies spanning general topics such as biological processes, molecular entities and scientific protocols and investigations, including a number of plant-focused ontologies.

While the OBO Foundry includes a number of plant-related ontologies, topics more relating to the plant sciences, such as plant genomics, phenomics, or agronomy domains are not the focus and lack the same level of community involvement as the biomedical domain ontologies. For this reason, a number of ontology repositories specifically geared to different facets of the plant sciences have been developed. Among these is AgroPortal, a vocabulary and ontology repository founded by the Montpellier scientific community to facilitate open and collaborative science in agronomy (http://agroportal.lirmm.fr/; Jonquet et al., 2018). AgroPortal was specifically designed for agronomy and related domains and reuses the openly available OntoPortal software (https://ontoportal.org/; Jonquet et al., 2023) to build the repository and services platform. The project aims to provide a reliable service involving hosting, searching and improving ontologies, allowing users to actively participate in the platform by uploading content and commenting on others’ content (Jonquet et al., 2018). The original motivation for AgroPortal was guided by five agronomic use cases, which cover a range of agronomic topics from germplasm to livestock and contribute to the design and focus of the portal (https://agroportal.lirmm.fr/about). These use cases include the Agronomic Linked Data knowledge base (http://agrold.southgreen.fr/aldp/; Venkatesan et al., 2018), a knowledge-based database for plant molecular networks such as genes, proteins, metabolic pathways and plant traits, and the Crop Ontology (CO) Project (https://cropontology.org/; Arnaud et al., 2022) of the Integrated Breeding Platform (https://www.integratedbreeding.net/), described in greater detail below. AgroPortal includes projects, vocabularies and ontologies which cover the entire range of agronomic research, from livestock and plant species to environmental conditions and land governance.

The Planteome database is heavily based on ontologies and is an informative resource for scientists searching for terminologies applicable to plant research and describing plant traits and experiments (https://planteome.org/). The database contains a collection of general reference ontologies aimed at improving annotation of an array of plant-related research data, ranging from genes to phenotypes (Cooper et al., 2018). Planteome also actively maps the species-specific ontologies of the Crop Ontology against the species-neutral reference ontologies, allowing users to search for a trait without having to consider the specific species. This is particularly useful for studies in comparative genomics or investigations of a family or clade (Cooper et al., 2018). As with the previously mentioned platforms, the Planteome ontologies are publicly available and openly maintained via GitHub repository (https://github.com/Planteome) to encourage sharing, tracking of revisions and new term requests (Cooper et al., 2018).

A number of additional repositories for finding and querying ontologies are also available and include Ontobee (https://ontobee.org/; Ong et al., 2017), BioPortal (https://www.bioontology.org/; Whetzel et al., 2011), the Ontology Lookup Service (https://www.ebi.ac.uk/ols/index; Côté et al., 2006), and the newly developed TIB Terminology service (https://terminology.tib.eu/ts; Strömert et al., 2023). While their collections do not focus heavily on the plant sciences, all aim to facilitate data sharing, ontology visualization, querying, integration and analysis. As the plant sciences cover a wide range of different technologies, many of the ontologies collected in these repositories will contain terms relevant to experimental set up and analysis. For example, all repositories listed above contain the Chemical Methods Ontology (CHMO) (https://github.com/rsc-ontologies/rsc-cmo), an ontology developed to describe chemical methods applicable to experimental assays, such as electron microscopy, preparations of materials to be separated for further analysis, such as by electrophoresis, and the synthesis of materials. Terms included in CHMO are relevant to many common techniques used throughout plant-related experiments, making it crucial the annotation of metadata for plant-related research.

## 4 The landscape of ontologies for fundamental plant science

**Figure 2:**
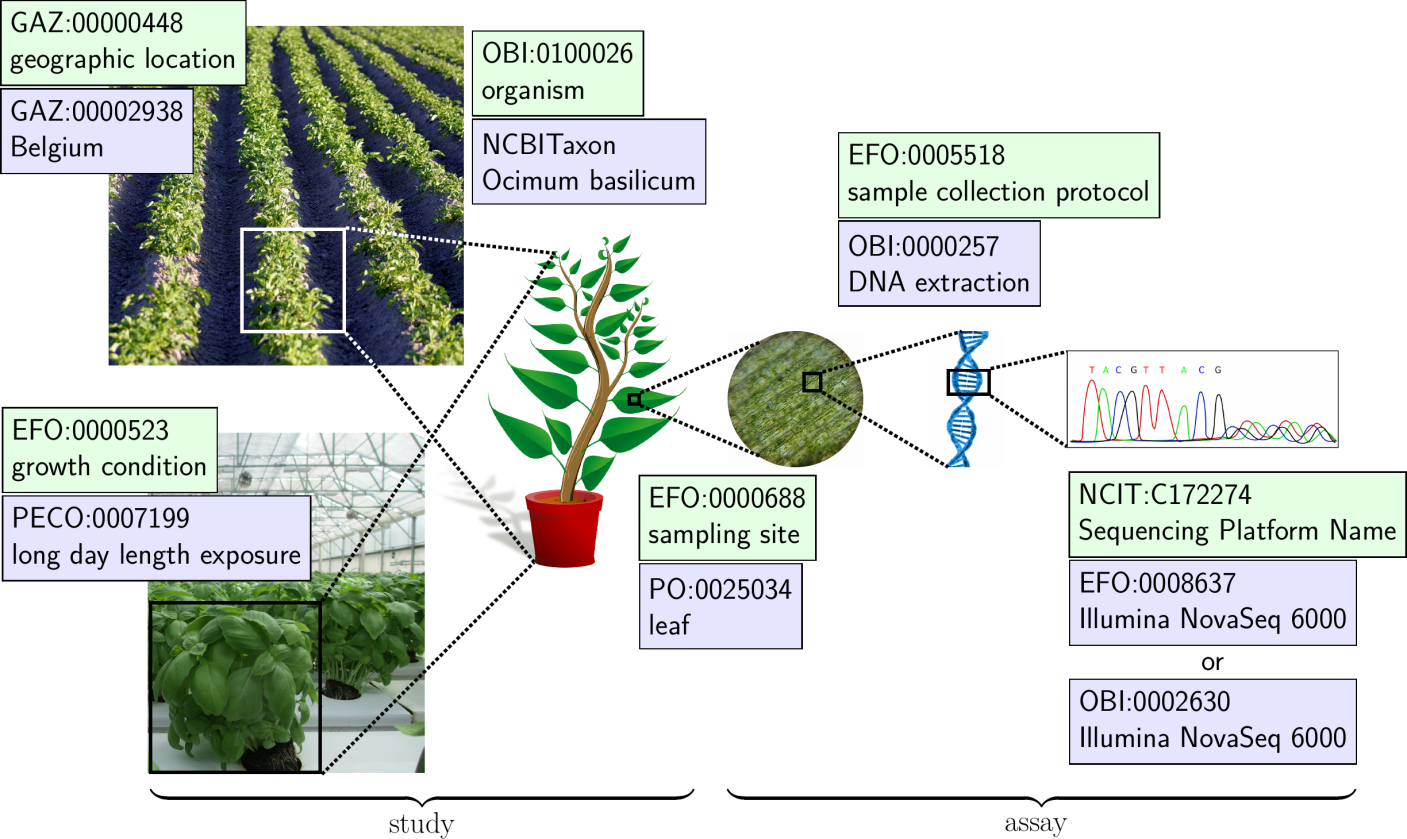
An example of how ontologies can be used for annotating a plant science experiment to increase the FAIRness of data. Terms must adequately cover the location and condition under which the plants are grown, the samples collected, and how the samples were processed and analyzed. For this reason, a diversity of ontologies is required to capture the multidimensional nature of the experiment. Also shown are the ISA metadata sections describing the growth and sample preparation protocols and samples in the *Study* and the protocols and research data related to an *Assay*.

One challenge of identifying ontologies relevant to fundamental plant research is the diverse range of methods and technologies that can be and are utilized for an average investigation or research project. The mechanisms governing processes such as plant development or resistance to stress and disease are complex and oftentimes a variety of techniques spanning different scientific fields are required to comprehensively elucidate these pathways and responses. For example, physiological changes such as photosynthesis, chemical changes to the soluble leaf fraction, and changes in gene expression of target pathways must all be combined to comprehensively characterize how progressive soil drought stress influences sugar alcohol accumulation in soybean (Dumschott et al., 2019). To properly annotate the metadata accompanying such a study, ontology domains covering plant traits, experimental conditions, experimental protocols, equipment and technologies, and measurement units must all be incorporated (**Figure 2**). In this section, we will outline the ontologies most relevant to the fundamental plant sciences, divided into two subsections: general scientific ontologies and plant-related ontologies. A list of relevant ontologies and their domains can be found in **Table 1**. An extended version is available in **Supplementary Table S1**.

**Table 1:** Commonly used ontologies for plant science research.

Ontology ID	Ontology Name	Domain	References
Plant-specific ontologies			
AGRO	AGRonomy Ontology	Agronomic practices, techniques, and variables used in agronomic experiments.	Aubert et al., 2017
CO	Crop Ontology	Breeder’s traits and variables	Shrestha et al., 2012; Arnaud et al., 2016; Arnaud et al., 2022
FLOPO	Flora Phenotype Ontology	Traits and phenotypes of flowering plants	Hoehndorf et al., 2016
PECO	Plant Experimental Conditions Ontology	Plant experimental conditions	Cooper et al., 2018
PO	Plant Ontology	Plant anatomy, morphology and growth and development	Walls et al., 2012; Cooper et al., 2013; Walls et al., 2019
PPEO	Plant Phenotype Experiment Ontology	Plant Phenotypes and Traits (implementation of the Minimal Information About Plant Phenotyping Experiment)	Papoutsoglou et al., 2020
PPO	Plant Phenology Ontology	Phenology of individual plants and populations of plants	Stucky et al., 2018
PSO (OBO Foundry and OLS)	Plant Stress Ontology	Biotic and abiotic stresses that a plant may encounter	Cooper et al., 2018
TO	Plant Trait Ontology	Phenotypic traits in plants	Cooper et al., 2018
General scientific ontologies – Domain-specific			
BAO	BioAssay Ontology	Biological screening assays and their results	Abeyruwan et al., 2014
BCO	Biological Collections Ontology	Support the interoperability of biodiversity data, including data on museum collections, environmental/metagenomic samples, and ecological surveys	Walls et al., 2014
BOF	Biodiversity Ontology	Biodiversity, as developed by the National Institute for Amazonian Research	Albuquerque, 2011
BTO	BRENDA Tissue and Enzyme Source Ontology	Source of an enzyme comprising tissues, cell lines, cell types and cell cultures	Gremse et al., 2011
CHEBI	Chemical Entities of Biological Interest Ontology	Molecular entities of biological interest	Degtyarenko et al., 2008
CHMO	Chemical Methods Ontology	Methods used to collect data in chemical experiments	https://github.com/rsc-ontologies/rsc-cmo
EDAM	EDAM Ontology of Bioscientific Data Analysis and Data Management	Computational biology, bioinformatics and bioimage informatics	Ison et al., 2013
EFO	Experimental Factor Ontology	Experimental variables	Malone et al., 2010
ENVO	Environment Ontology	Environmental systems, components and processes	Buttigieg et al., 2013; Buttigieg et al., 2016
GEOSPECIES	GeoSpecies Ontology	Integration of species concepts with species occurrences, gene sequences, images, references and geographical information	http://lod.geospecies.org/
GO	Gene Ontology	Function of genes and gene products	Ashburner et al., 2000; Gene Ontology Consortium et al., 2023
MMO	Measurement Method Ontology	Methods used to make clinical and phenotype measurements	Smith et al., 2013
MOD	Protein modification (PSI-MOD)	Protein chemical modifications, classified by molecular structure or amino acid	Montecchi-Palazzi et al., 2008
MS	PSI Mass Spectrometry Ontology	proteomics mass spectrometry	Mayer et al., 2013
MSIO	Metabolomics Standards Initiative Ontology (MSIO)	mass-spectrometry and nmr-spectroscopy based metabolomics experiments and fluxomics studies	Rocca-Serra, 2018
NCBITAXON	National Center for Biotechnology Information (NCBI) Organismal Classification	NCBI organismal taxonomy	Federhen, 2012
NCIT	National Cancer Institute Thesaurus	Broad coverage of the cancer domain	https://ncit.nci.nih.gov
OBI	Ontology for Biomedical Investigations	Life-science and clinical investigations	Brinkman et al., 2010; Bandrowski et al., 2016
PATO	Phenotype And Trait Ontology	Phenotypic qualities (properties, attributes or characteristics)	Gkoutos et al., 2018
PCO	Population and Community Ontology	Material entities, qualities, and processes related to collections of interacting organisms such as populations and communities	Walls et al., 2014
STATO	Statistics Ontology	statistical tests, conditions of application, and information needed or resulting from statistical methods	https://github.com/ISA-tools/stato
SWO	Software Ontology	software tools, their types, tasks, versions, provenance and associated data	Malone et al., 2014
UBERON	Uberon multi-species anatomy ontology	An integrated cross-species anatomy, covers animals and bridges multiple species-specific ontologies	Mungall et al., 2012
UO	Unit Ontology	Metrical units for use in conjunction with PATO	Gkoutos et al., 2012
General scientific ontologies – Upper-level ontologies			
BFO	Basic Formal Ontology	Standardizes upper-level structure of OBO ontologies	Arp et al., 2015
COB	Core Ontology for Biology and Biomedicine	Framework for building ontologies in the life sciences	https://github.com/OBOFoundry/COB
RO	Relation Ontology	Standardizes relations in OBO Foundry ontologies	https://oborel.github.io/obo-relations/

While in most cases, the ontology term ID space is the same across different portals, in a few cases e.g. OBO Foundry and BioPortal use different short names. An extended version is available in **Supplementary Table S1**.

### 4.1 General scientific ontologies

General scientific ontologies cover concepts, relationships, and properties within a particular domain that can be applicable to wider scientific fields. We identified both upper-level and domain-focused ontologies as being relevant and important for the annotation of research data within the fundamental plant sciences. These ontologies provide a common vocabulary for representing and integrating scientific knowledge from different scientific domains, covering topics such as experimental set-ups or measurement units used, or else provide a framework for unifying ontologies for increased interoperability. In this section, we outline examples of general scientific ontologies, both domain-specific and upper-level ontologies, that are most relevant to contemporary fundamental plant research.

Upper-level ontologies provide a foundational framework for other ontologies to incorporate in order to create semantic interoperability (Hoehndorf, 2010). As they are independent of any particular domain or application and provide the most general concepts, they can be easily integrated into ontologies and facilitate data integration across different systems and domains (Mascardi et al., 2007, **Figure 1C**). We identified the Basic Formal Ontology (BFO), the Core Ontology for Biology and Biomedicine (COB), and the Relation Ontology (RO) as being upper level ontologies most relevant to the fundamental plant sciences. BFO provides a framework for building more specific domain ontologies. It aims to provide a common foundation of concepts and relationships that can be used to represent knowledge in a wide range of domains such as biology, biomedical informatics, natural language processing, and philosophy (Arp et al., 2015). COB is a basic and structured framework that serves as a foundational resource for the life sciences (https://github.com/OBOFoundry/COB). By capturing essential concepts and their relationships, it serves as a foundation for the development of advanced ontologies for biology and biomedicine (Abdelmageed et al., 2021). A general scientific ontology that defines all properties used by the OBO Foundry ontologies is the RO. It is a formal ontology that provides a framework for interoperability between different ontologies and can therefore be used for different contexts (Smith et al., 2005).

One crucial aspect of successfully understanding the context of a dataset, is understanding how an experiment was performed. Variations in what treatment was performed on a sample, what protocols were used to extract a target material, or how a sample was collected must all be properly annotated for the resulting data and analysis to have any meaning. For this reason, the Ontology for Biomedical Investigation (OBI) (Brinkman et al., 2010) and the Experimental Factor Ontology (EFO) (Malone et al., 2010) were developed. Although it has ‘biomedical’ in the name, OBI is an integrated ontology for the description of all aspects of life-science investigations, even in the plant sciences, covering all phases of investigations, from planning to reporting (Bandrowski et al., 2016). OBI reuses terms from other well-established ontologies, in order to cover the wide range of projects it is intended for. It defines terms for assays such as mass spectrometry assay (OBI:0000470), instruments such as chromatography device (OBI:0001139), objectives and other concepts needed for data collection and analysis, including those involving omics and multi-omics approaches. As the methods used in the plant sciences encompass a wide range of technologies, researchers will find many relevant terms described in OBI. EFO is developed and maintained by the European Bioinformatics Institute (EMBL-EBI) to support the annotation, analysis and visualization of data handled by groups involved in the organization. Although focused on gene expression data (Malone et al., 2010), describing experimental factors requires concepts taken from many disciplines, ranging from cell biology and proteomics to anatomy and environmental science. There are many terms within EFO that are relevant to describing sample collection and experimental factors within plant science investigations. For example, EFO contains terms that can help annotate the conditions under which a plant was grown (growth condition, EFO:0000523) (**Figure 2**) or else what part of a plant was sampled for an analysis (sampling site, EFO:0000688) (**Figure 2**). It is important to note that there are overlaps in terms between OBI and EFO. For example, both ontologies contain terms describing instruments such as Illumina NovaSeq 6000 (OBI:0002630, EFO:0008637) (**Figure 2**). Deciding on which term to use comes down to the personal preference of the researcher or else common practices within the research community.

When annotating investigations involving molecular pathways and the characterization of gene expression and function, ontologies such as the Gene Ontology (GO) (Ashburner et al., 2000) and MapMan (Schwacke et al., 2019) are good resources to consider. GO is a widely used standardized classification system that provides a controlled vocabulary and framework for describing the functions, processes, and cellular components associated with genes and gene products, such as proteins, across different species (Ashburner et al., 2000; Gene Ontology Consortium et al., 2023). It helps researchers annotate and interpret genomic data by assigning functional terms to genes based on experimental evidence and computational predictions. Terms within the ontology are divided into three main categories: biological processes, molecular function and cellular component. Terms are connected within these categories, forming directed acyclic graphs. GO enables the comparison of gene function across diverse organisms, facilitates data integration and analysis, and supports the discovery of new biological insights by providing a structured and comprehensive framework for studying gene functions in the context of biological systems.

In contrast to GO, the MapMan4 ontology was developed specifically for the characterization of gene expression and biological functions in plants (Schwacke et al., 2019). Built upon the original MapMan framework (Thimm et al., 2004), MapMan4 represents common biological processes and genetic information gathered from a wide range of plant species. It is organized in a tree structure, with top levels being main biological concepts and subsequent sublevels becoming more specialized to ensure the most precise protein characterization possible. The tool Mercator is used for the automatic annotation of protein sequences with the MapMan4 ontology (Schwacke et al., 2019).

As the field of fundamental plant research incorporates many different scientific domains, there are a number of ontologies in other natural and life-science disciplines that are often applicable for a plant science investigation, depending on the topic and scope. Chemical Entities of Biological Interest (ChEBI) (Degtyarenko et al., 2008) and the Environment Ontology (ENVO) (Buttigieg et al., 2013; Buttigieg et al., 2016), covering chemical and environmental aspects, respectively, are two such often-utilized, cross-discipline ontologies. Developed by EMBL-EBI, ChEBI provides a classification of chemical entities (Degtyarenko et al., 2008). The ontology can be divided into three main branches: chemical entities, the role the chemical entity can have, and subatomic particles. Its importance to the wider scientific community is evident as ChEBI is widely incorporated into various databases, such as UniProt (The UniProt Consortium, 2015), and is heavily reused in well-established ontologies, such as GO, OBI, and EFO, as well as plant-specific ontologies. Within the plant sciences, ChEBI contains many terms for metabolites commonly found in plants, including primary metabolites such as glucose (CHEBI:17234) and fructose (CHEBI:287570), secondary metabolites such as pinitol (CHEBI:372080), and a wide range of terms for carotenoids (CHEBI:23044 and child terms). For environmental entities, ENVO is a widely-used ontology for describing ecosystems, environmental processes or even entire planets. Originally designed to provide information regarding biomes and environmental features of genomic and microbiome samples for the Genomics Standards Consortium (Field et al., 2011), ENVO has since evolved into a cross-discipline resource, spanning domains from biomedicine and omics to anthropogenic ecology and socioeconomic development (Buttigieg et al., 2016). As ENVO evolved, developers implemented changes to better align to OBO Foundry principles to ensure increased interoperability, such as incorporating terms from RO and BFO and moving the ontology to its own GitHub repository for better version control.

Finally, there are a number of ontologies specifically relating relevant metadata important for describing the measurement of samples and subsequent data analysis. For example, the Human Proteome Organization- Proteomics Standards Initiative (HUPO-PSI) developed the PSI-Mass Spectrometry (MS) controlled vocabulary to logically structure and capture all terms relating to an MS pipeline- from sample preparation (in-solution digestion, MS:1002986) to instrument models (LCMS-9030, MS:1002998), parameters (Mascot : SigThreshold, MS:1001316) and related software (Spectronaut, MS:1001327) (Mayer et al., 2013). Examples of ontologies for the annotation of software used during the analysis of data collected within the life sciences are the Software Ontology (SWO) (Malone et al., 2014) and the Ontology of Bioscientific Data Analysis and Data Management (EDAM) (Ison et al., 2013). The need for such ontologies was realized as bioinformatic analysis became ever more prevalent. Just as metadata annotation is crucial for the reproducibility of laboratory experiments, knowing what versions of what tools were used to analyze a dataset is necessary for the reproducibility of said analyses. The scope of SWO is broad as it incorporates tools and software versions not only used in bioinformatics analyses (SAMtools, SWO:1100143), but also tools (and their versions) used for the management, analysis and presentation of biological data (R software, SWO:1100075) (Malone et al., 2014). EDAM covers topics, operations, types of data and data identifiers (Database ID, EDAM:1048) and formats (RNA annotation format, EDAM:3824) relevant to data analysis and management in the life sciences (Ison et al., 2013).

While a number of ontologies and their applications have been described above and in **Table 1**, they are just a small subset of ontologies available to fundamental plant scientists. Depending on the particular investigation and technologies being employed, researchers may require an ontology that covers a domain not covered in this review, like food and nutritional ontologies. For this reason, it is always recommended to consult established ontology repositories to find a term or ontology that most closely matches the metadata being annotated.

### 4.2 Plant-specific ontologies

There are several ontologies well suited for describing and annotating experiments, phenotypic traits, structures and experimental conditions relating to plant research. In the following section, we will describe the most relevant ones in greater detail.

#### 4.2.1 The Planteome project reference ontologies

The Planteome project (https://planteome.org/; Cooper et al., 2018) develops and maintains a number of species-neutral reference ontologies, including the Plant Ontology (PO) (Walls et al., 2012; Cooper et al., 2013), the Plant Trait Ontology (TO) (Cooper et al., 2018) and the Plant Experimental Conditions Ontology (PECO) (Cooper et al., 2018). The PO is crucial for the consistent annotation of anatomy, morphology and developmental stages of both plants and plant parts (Walls et al., 2012). Originally focused on *Arabidopsis thaliana* (mouse-ear cress) *Zea mays* (corn) and *Oryza sativa* (rice), it was broadened to cover all Viridiplantae (green plants). The primary aim of the PO is to bridge the diversity of data that can be collected during plant research- from genetics, molecular and cellular biology to taxonomy and botany research (Walls et al., 2019). The PO is divided into two main branches: ‘plant anatomical entity’ and ‘plant structure development stage’. Terms in these branches are organized hierarchically via subclass, or subClassOf (is_a) relations. All other relations depicted in the PO come from the OBO RO (Walls et al., 2019). The branch ‘plant anatomical entity’ includes terms for plant morphology and anatomy, such as structures (leaf, PO:0025034) (**Figure 2**) (Walls et al., 2012), whereas the branch ‘plant structure development stage’ covers terms relating to stages of life either of a whole plant or plant part during which the structure undergoes developmental processes, such as growth (rosette growth stage, PO:0007113), differentiation (root cortex differentiation stage, PO:0007513) or senescence (sporophyte senescent stage, PO:0007017) (Walls et al., 2019).

Two other plant reference ontologies used in the Planteome database are the TO and PECO. Both ontologies were conceived and developed with the aim of improving data interoperability for the advancement of plant research. One common issue within the fundamental plant sciences is the semantic inconsistencies that exist between species, especially for phenotypic descriptions, meaning data integration is often not possible without the manual identification of corresponding concepts (Arnaud et al., 2012). For example, what is referred to as a ‘leaf’ in some species is known as a ‘frond’ in others. It is therefore crucial to standardize trait terms between different species and projects in a way that allows for the easy integration of phenotypic and trait data from different sources. The TO was developed to address the discrepancies in trait descriptions and to increase interoperability of plant trait data between species (Cooper et al., 2018). Terms within the TO are structured according to an Entity-Quality pattern (Arnaud et al., 2012). Entity terms are imported from other well-established ontologies, such as the PO, the GO and ChEBI, while quality terms are taken from the Phenotype and Trait Ontology (PATO). In this way, terms and descriptions are kept general enough that they can be successfully applied to most plant species and importing terms from other ontologies facilitates its interoperability. For example, the term biological process trait (TO:0000283) is a subclass of the PATO term process quality (PATO:0001236), and subclasses of biological process trait include terms such as fruit ripening trait (TO:0000929), net photosynthetic rate (TO:0001027) and starch grain synthesis (TO:0002658). Finally, PECO covers terms specifically needed to describe study types (greenhouse study, PECO:0007248), growth conditions (long day length exposure, PECO:0007199) (**Figure 2**) and treatments assessed during an experiment, including abiotic treatments such as sodium chloride exposure (PECO:0007048), or biotic treatments, such as arbuscular mycorrhizal fungal exposure (PECO:0001059) (Cooper et al., 2018).

#### 4.2.2 The Crop Ontology

The Crop Ontology (CO) was developed by several members of the Consultative Group on International Agricultural Research (CGIAR) to harmonize the annotation of phenotypic and genotypic data between different crops (https://alliancebioversityciat.org/tools-innovations/crop-ontology). As traits, measurement methods and scales can vary greatly between different crops, controlled vocabularies and ontologies are required to enable comparisons between how a trait is assessed in different species (Shrestha et al., 2012). The CO provides crop-specific trait ontologies for increased plant data annotation and integration (Shrestha et al., 2012; Arnaud et al., 2016; Arnaud et al., 2020). Terms are cross-referenced with synonyms in PO and TO, increasing the interoperability of data as users are able to search for a trait without having to consider a specific species (Arnaud et al., 2012). This feature is important for studies in comparative genomics or when searching for traits shared by a family or clade of plants (Cooper et al., 2018). At the time of writing, 37 species-specific ontologies are included, covering a wide range of different crop species, including staple crops (wheat, maize, cotton, soybean), fruits and vegetables (banana, brassica) and legumes (chickpea, mung bean, lentil, faba bean).

#### 4.2.3 Plant Phenology Ontology

While a number of large continental-scale data sources for plant phenology exist, the ability to conduct analyses of plant phenology on an inter-continental scale was hindered by the lack of standardized language and terminology used to describe the data found in individual repositories, resulting in data incompatibility. For this reason, the Plant Phenology Ontology (PPO) was developed to address this communication gap and help to facilitate interoperability of plant phenology data (Stucky et al., 2018). Six principles guide the design and goals of the PPO to ensure it is both broadly applicable as well as interoperable and that data based on PPO annotation is reusable. The PPO aims to reuse terms from other ontologies, such as the PO and Biological Collections Ontology (BCO) wherever possible (Walls et al., 2014). The classes and concepts included in the PPO can be divided into three main topics: plant structures, phenological traits and observations of/data about phenological traits (Stucky et al., 2018).

#### 4.2.4 The Plant Phenotype Experiment Ontology

One of the most challenging fields of fundamental plant research is that of phenotyping, due to its heterogeneous nature and the sensitivity of phenotype to environmental conditions. The ability to correctly interpret phenotypic data is therefore heavily reliant on how completely environmental conditions and metadata relating to experiment setups is recorded (Papoutsoglou et al., 2020). Therefore, Ćwiek-Kupczyńska et al. (2016) created the “Minimum Information About a Plant Phenotyping Experiment” (MIAPPE) to outline the list of attributes, or metadata, necessary to adequately annotate a plant phenotyping experiment so that the resulting data can be correctly understood. Attributes included within MIAPPE are organized into different sections according to the ISA framework (described in more detail in Section 5) as it is able to handle a diverse range of phenotyping data and experimental designs due to its generality and flexibility (Ćwiek-Kupczyńska et al., 2016). To facilitate the implementation of MIAPPE, the Plant Phenotyping Experiment Ontology (PPEO) was created. Whereas most of the plant-specific ontologies described above focus on defining terms for metadata description, PPEO represents the MIAPPE structure, incorporating the different sections of the framework as the primary backbone of the ontology, then adding the required attributes (https://github.com/MIAPPE/MIAPPE-ontology). PPEO is therefore an important resource for organizing metadata collected during plant phenotyping experiments (rather than defining metadata terms), as all attributes have their own ontology id, making them easily searchable.

#### 4.2.5 The DataPLANT Biology Ontology (DPBO)

The NFDI funded DataPLANT consortium has developed its own ontology, the DataPLANT Biology Ontology (DPBO) (https://github.com/nfdi4plants/nfdi4plants_ontology), to assist researchers with metadata annotation and help close the ontology gap. In conjunction with a collection of external ontologies, the DPBO helps users annotate their experimental metadata via DataPLANT’s Swate tool (https://github.com/nfdi4plants/Swate; Mühlhaus et al., 2021). The DPBO contains terms from established ontologies needed by users as well as new terms not yet found in any ontology. Users suggest the terms they need via the GitHub issues tab and the DPBO curators provide quick feedback to term suggestions and incorporate them into the DPBO. For new terms, curators find the most fitting ontology and suggest the terms to be incorporated. In this way, researchers can use their needed terms for metadata annotation without having to go through the time and effort to select the most relevant ontology and DataPLANT acts as the middle man between researchers and ontology providers. Once a term has been accepted to the external ontology, the DPBO term is deprecated with a reference to the new term id. Thus, the DPBO provides a low-friction way for researchers to contribute to closing the ontology gap.

## 5 Implementation of metadata frameworks for ontology-enriched metadata annotation

Challenges in data harmony and integration often arise when bringing together data from multiple sources to answer complex scientific questions. These challenges involve differences in terminology descriptions, a lack of sufficient context, or else the data is structured in a way being difficult to comprehend, thereby hindering its reuse. To address these challenges and facilitate the management and integration of experimental data across different research domains, a number of community-driven efforts have been founded that aim to develop metadata standards and frameworks for improved data sharing and handling. Some well-known examples are Bioschemas (Michel and The Bioschemas Community, 2018) and the ISA (Investigation-Study-Assay) framework (González-Beltrán et al., 2014; Sansone et al., 2016).

**Figure 3:**
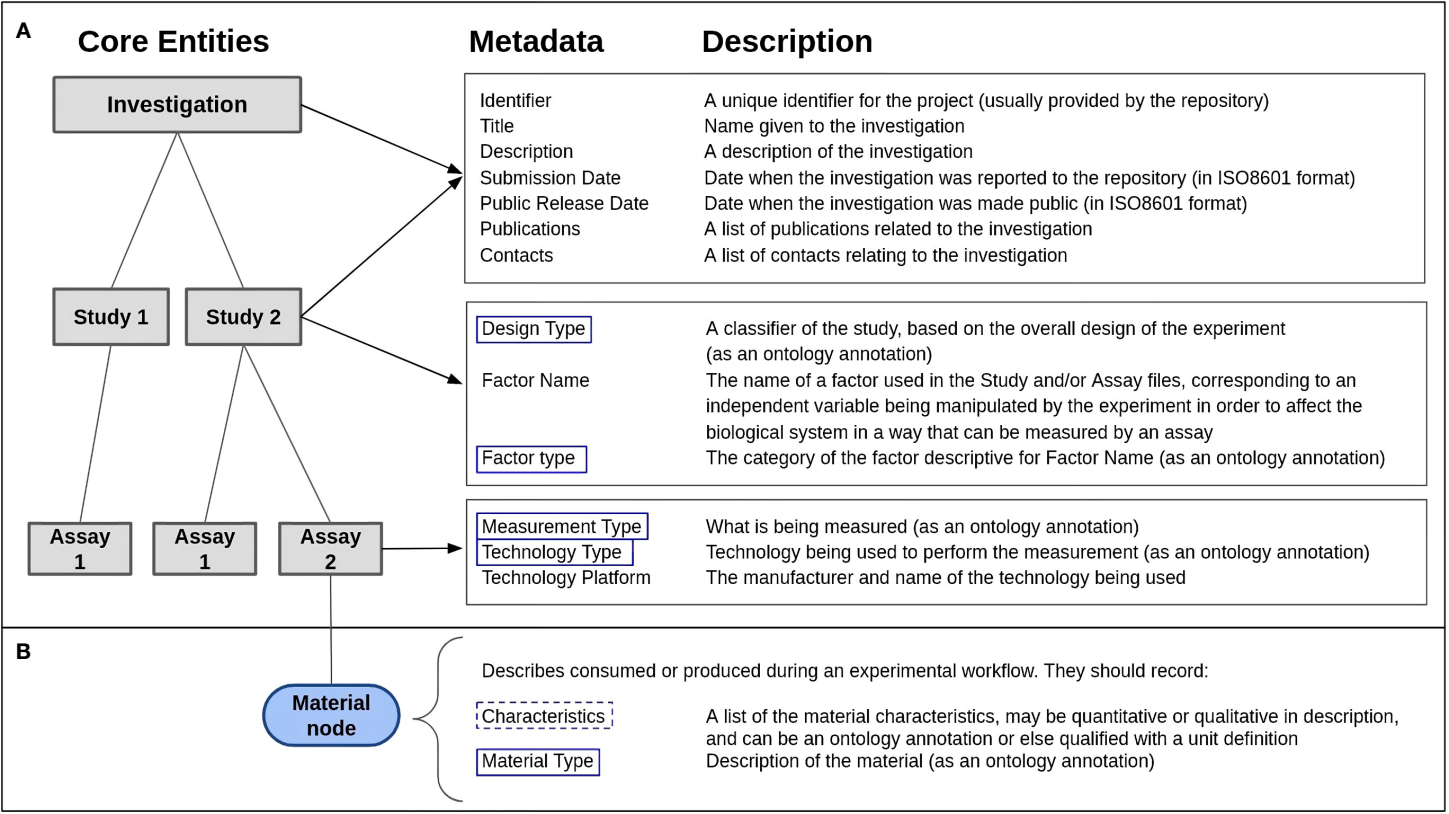
The ISA metadata framework (Sansone et al., 2016), designed to capture experimental metadata. **(A)** The framework consists of three core entities: *Investigation*, *Study* and *Assay*. The structure allows for multiple studies to be described within one investigation and multiple assays to be described within one study. Each entity has metadata requirements that should be included to ensure the complete description of the entity. Within the *Study* and *Assay* entities, an ontology annotation is the required input for included metadata, such as design type in study and measurement type in assay (marked with blue boxes). **(B)** An example of the ‘material node’ that *Assay* entities can contain to describe material consumed or produced during an experiment. The required metadata for this node are ‘characteristics,’ which may-but does not have to be- an ontology annotation (marked with a dashed blue box) and ‘material type,’ which required an ontology annotation (marked with a blue box).

The ISA framework aims to ensure scientific data is accompanied by metadata that describes the context in which the data was generated. This context includes information about the experimental design, sample characteristics, data acquisition and processing, and data analysis. The ISA data model is structured around three entities: Investigation, Study, and Assay (**Figure 3**). The *Investigation* entity represents the overarching research project and provides general information about the research questions and goals. The *Study* entity describes the subject under study and the experimental design. For a plant study, this could include factors and protocols with respect to growth of the plants or generation of samples of plant origin, either parts of a plant or the plant itself. The *Assay* entity represents the measurements taken on the collected sample as well as the data generated from those measurements, including raw and derived data files. For a plant assay, this could include protocols on how a sample was processed, such as for a DNA extraction, and information on the measuring instrument used (i.e. sequencer used to process the extraction sample).

One advantage of the ISA framework is its flexibility and adaptability, which allows for a wide range of experimental designs and research data to be represented within a single investigation. Its flexibility is exemplified by the ability to gather metadata in a user-friendly spreadsheet format, which is not only human-readable but also effortlessly convertible into machine-readable formats essential for numerous applications and software systems (e.g. ISA-Tab, ISA-JSON etc.) (González-Beltrán et al., 2014). Different types of biological data, such as transcriptomic, proteomic, or metabolomic data, as well as non-biological data, such as environmental conditions, can be represented by the ISA model (Sansone et al., 2012).

Ontologies play a crucial role in the ISA data model by providing a standardized vocabulary and sets of concepts that can be used to describe experimental metadata in a consistent and structured way. Each ISA entity has a list of required metadata, where ontology annotations are preferred over free-text to give the proper context for the entity (**Figure 3A**). According to the ISA specification, each entity has additional nodes to describe material or data related to the experiment, where ontology terms can further be annotated. For example, under the *Assay* entity is the ‘material node’, where researchers can describe materials consumed or produced during an experimental workflow. Within this node is the property “material type”, which should be described using an ontology annotation (**Figure 3B**). This makes the description of experimental metadata both precise and unambiguous, reducing the potential for errors and misinterpretation, enabling data to be more easily integrated and shared across different research projects, domains and platforms. Ontologies can also be used to map the relationships between different concepts and terms, which can facilitate potential connections and correlations between different datasets.

**Figure 4:**
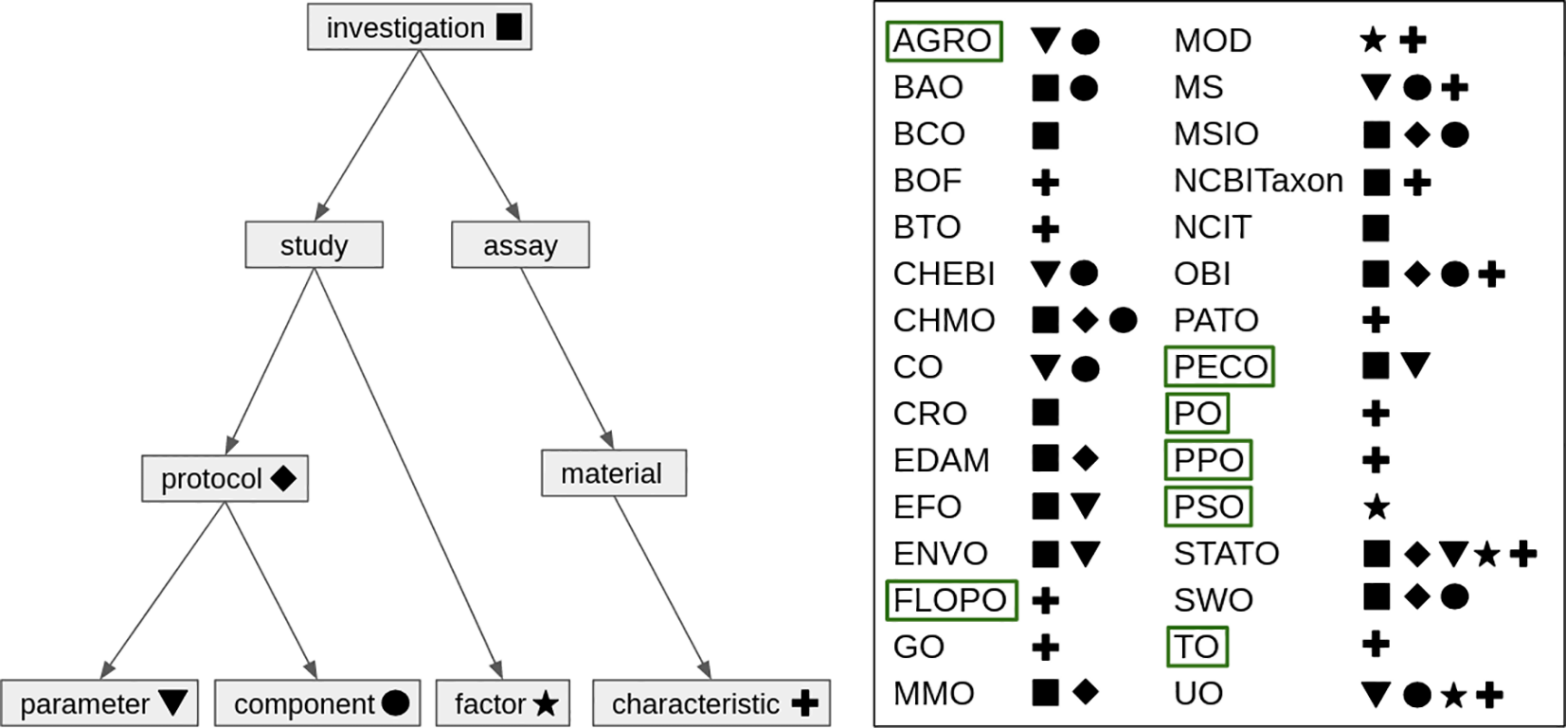
Visualization of the domain-specific ontologies relevant for the plant science community and their relation to ISA. Left: hierarchical structure of ISA concepts. The ontologies on the right are linked to ISA concepts via symbols. Green boxes highlight plant-specific ontologies. While this figure gives an idea of where ontologies can be incorporated within ISA, there are likely scenarios where an ontology can be incorporated that is not depicted here.

There are a number of ways that plant-related ontologies can be incorporated into one of the ISA entities depending on the investigation that is being represented (**Figure 4**). For example, OBI and EFO can be incorporated into the *Study* and *Assay* entities of the ISA framework as they contain terms relating to experimental setup, sample processing and analysis. ENVO is particularly relevant for annotating *Study* metadata as terms can be used when describing the habitat or environmental conditions of a location when a plant sample was collected (for example, ENVO_1000745: drought).

One of the key benefits of using ontologies in the ISA data model is that it allows for more effective data integration and analysis, by controlling the values that a metadata element can take (for example Ho Sui et al., 2013; Peters et al., 2019). Using ontologies to standardize the terms used to describe experimental variables allows researchers to more easily compare datasets and identify similarities and differences between them. This can be especially important when analyzing large datasets, where manual inspection and interpretation of the data may be difficult or time-consuming. In addition to providing a standardized vocabulary, ontologies also help to improve the accuracy and consistency of the data itself. By using an ontology to specify the units of measurement used in an experiment, researchers can avoid errors that might arise from using different units or from converting units incorrectly. This can be especially important when working with complex datasets that involve many different types of measurements and units.

Overall, ontologies are an important tool for enhancing the effectiveness and efficiency of the ISA data model (Johnson et al., 2021). By providing a standardized vocabulary and set of concepts, ontologies help to ensure that the experimental metadata is precise, consistent, and easy to interpret and share across different research projects and domains.

## 6 Discussion and perspectives

As the potential of integrating modern techniques becomes evident, the need to properly integrate and manage the data produced becomes evermore important. Ontologies play a crucial role in this management, ensuring that data is both reusable and interoperable by ‘tagging’ data. Tagged data is then both human- and machine-readable, allowing for the subsequent retrieval and standardization of data. Incorporating terms into metadata frameworks, such as ISA, further increases the FAIRness of data. However, despite the obvious advantages of incorporating ontologies into data management schemes, determining what ontology or ontological term is most appropriate at any given time can be challenging due to the confusing and overlapping nature of the ontological landscape. For example, both OBI and EFO include terms for sequencing instruments (**Figure 2**, Section 4.1) and either ontology can be selected for annotation. The reality is that there is no ‘one size fits all’ approach to what terms should be used to annotate metadata, and it is often a matter of familiarizing oneself with what ontologies and terms are available. Fortunately, there has been a push in recent years to provide more comprehensive overviews of ontologies that are available to the wider scientific community. A number of ontology repositories have been developed, all aiming to facilitate researchers in finding terms most fitting for annotating metadata. Ontology repositories and service platforms such as Planteome and AgroPortal are good resources for users trying to determine where to begin when annotating fundamental plant research data with ontology terms. Both are designed specifically for plant research, agronomy and related fields and encourage the active participation and collaboration of users for the improvement of included ontologies. As the number of agricultural genetics, genomics and breeding databases increases, the need for better data and metadata sharing will too, and ontology use and promotion of data standards will grow (Clarke et al., 2023).

Another potential challenge facing users is where to actually begin with when it comes to metadata annotation as it is not always clear what should be included when describing an experiment. This challenge has been recognized by the wider scientific community and a number of resources, tools, and projects have been developed in recent years to address these hurdles. Minimum information standards, such as MIAPPE (Ćwiek-Kupczyńska et al., 2016), often provide comprehensive lists of required, recommended and optional attributes needed for the complete description of an experiment. Tools such as DataPLANT’s Swate (https://github.com/nfdi4plants/Swate; Mühlhaus et al., 2021) and ISA’s OntoMaton (Maguire et al., 2013, https://github.com/ISA-tools/OntoMaton) can be utilized to help users when beginning the process of annotating their spreadsheets with ontology terms. Swate has a number of templates available that can be used when submitting to repositories or if the researcher wants their data collection to comply with a minimum information standard. A number of recent collaborative projects have also recognized the need for better metadata management for the submission to repositories. For example, a data brokering prototype developed at the 2022 European Biohackathon provides a high-level alignment of ISA-JSON to the ENA (European Nucleotide Archive) XML submission template with the hope of streamlining the submission process (D'Anna et al., 2023), with future plans to expand to other repositories (https://github.com/elixir-europe/biohackathon-projects-2023/tree/main/27).

One common theme apparent throughout the ontologies covered within this review is the importance of open and collaborative efforts for increasing the FAIRness of data. It is widely accepted by the greater scientific community that making data more FAIR will greatly benefit data reproducibility and data management practices (Rocca-Serra et al., 2023). However, the actual task of making data FAIR remains costly and time consuming, hindering progress. Ontology providers therefore rely on and encourage community engagement and interdisciplinary collaboration for the continuing expansion and improvement of concepts and their relations. Through technologies such as Git, coupled with open licensing and standardized ontology languages, ontologies such as EFO (https://github.com/EBISPOT/efo), OBI (https://github.com/obi-ontology/obi), the Planteome ontologies (https://github.com/Planteome) and many others encourage communication and collaboration with researchers to help fill ontology gaps. Fundamental plant research is diverse by nature, incorporating techniques and concepts from all corners of the life sciences, and benefits greatly from this community push for the standardization and improvement of ontologies for metadata annotation.

The evolving nature of science requires that data can be reevaluated and benefits from a flexible data structure. For this reason, efforts such as NFDI (https://www.nfdi.de/; Hartl et al., 2021) and Elixir (https://elixir-europe.org/; Crosswell and Thornton, 2012) have been established in recent decades. These efforts recognize the importance of well-curated and managed datasets for the increased management and archiving of research data. One potential outcome of these efforts is the possibility of creating integrated repositories and enabling semantic searches upon those, facilitating the reuse of datasets to answer novel research questions. Similar work is already being done within the biomedical sciences with the development of resources such as GenoSurf (http://www.gmql.eu/genosurf/; Canakoglu et al., 2019), specific to human genetics. Databases such as the European Bioinformatics Institute’s BioSamples (https://www.ebi.ac.uk/biosamples/; Courtot et al., 2019) contains datasets from a wide range of different species, including many plant species. Also part of Elixir’s infrastructure, BioSamples contains results from biological samples stored in archives such as ArrayExpress (https://www.ebi.ac.uk/biostudies/arrayexpress; Kolesnikov et al., 2015), the European Nucleotide Archive (ENA) (https://www.ebi.ac.uk/ena/browser/home; Silvester et al., 2018) and the European Genome-phenome Archive (EGA) (https://ega.crg.eu/; Lappalainen et al., 2015). Specifically for plant data, the NFDI funded consortiums FAIRagro (https://fairagro.net/en/, Specka et al., 2023) and DataPLANT have developed their own infrastructure for data curation and archiving. The FAIRagro infrastructure contains a number of data repositories, including e!DAL-PGP (https://edal-pgp.ipk-gatersleben.de/; Arend et al., 2016), for the storage of data relating to agricultural research. DataPLANT has developed the PLANTdataHUB. Users have the option of submitting their datasets to the Annotated Research Context (ARC) registry, which provides an advanced search interface for locating relevant data via indexed metadata found within the datasets (Weil et al., 2023). With the recent push for sustainable RDM and FAIR data practices, the potential that ontologies provide to the plant research community becomes evermore apparent. However, knowing where to begin when annotating experimental metadata with ontologies is sometimes a daunting task. Here we have outlined ontologies most relevant to the fundamental plant sciences and discussed resources available for finding ontology terms. This review is meant as a starting guide for plant researchers when considering the metadata annotation of their next datasets. The complexity of both the ontology landscape and the data produced within plant experiments means that there is no ‘one size fits all’ approach to metadata annotation and makes a comprehensive guide impossible. Instead, researchers are encouraged to explore the resources outlined here and familiarize themselves with ontology terms to help them decide on the terms best suited to their individual experiments. The proper annotation of metadata with ontology terms will no doubt further good RDM practices, not only for the fundamental plant research community, but the wider scientific research community, making it the responsibility of every researcher to implement these annotations within their own research.

## Author contributions

KD: Conceptualization, Visualization, Writing – original draft, Writing – review & editing. HD: Visualization, Writing – review & editing. ML: Writing – review & editing. DB: Writing – review & editing. AS: Writing – review & editing. BU: Funding acquisition, Writing – review & editing. SN: Writing – review & editing. EA: Writing – review & editing. AK: Conceptualization, Writing – original draft, Writing – review & editing.
